# Identification of the Lomofungin Biosynthesis Gene Cluster and Associated Flavin-Dependent Monooxygenase Gene in *Streptomyces lomondensis* S015

**DOI:** 10.1371/journal.pone.0136228

**Published:** 2015-08-25

**Authors:** Chunxiao Zhang, Chaolan Sheng, Wei Wang, Hongbo Hu, Huasong Peng, Xuehong Zhang

**Affiliations:** State Key Laboratory of Microbial Metabolism, School of Life Sciences and Biotechnology, Shanghai Jiao Tong University, 800 Dongchuan Road, Shanghai, 200240, China; University Paris South, FRANCE

## Abstract

*Streptomyces lomondensis* S015 synthesizes the broad-spectrum phenazine antibiotic lomofungin. Whole genome sequencing of this strain revealed a genomic locus consisting of 23 open reading frames that includes the core phenazine biosynthesis gene cluster *lphzGFEDCB*. *lomo10*, encoding a putative flavin-dependent monooxygenase, was also identified in this locus. Inactivation of *lomo10* by in-frame partial deletion resulted in the biosynthesis of a new phenazine metabolite, 1-carbomethoxy-6-formyl-4,9-dihydroxy-phenazine, along with the absence of lomofungin. This result suggests that *lomo10* is responsible for the hydroxylation of lomofungin at its C-7 position. This is the first description of a phenazine hydroxylation gene in *Streptomyces*, and the results of this study lay the foundation for further investigation of phenazine metabolite biosynthesis in *Streptomyces*.

## Introduction

Natural phenazine compounds, a class of secondary metabolites containing a phenazine nucleus, are mainly produced by *Streptomyces* and *Pseudomonas* species [1,2]. Phenazines have many biological functions, and demonstrate antimicrobial, antifungal, anti-tumor, antimalarial, and antiparasitic activities. The phenazine compounds produced by *Pseudomonas* species usually have simple structures, such as phenazine-1-carboxyl acid (PCA) [3], 1-hydroxyphenazine [4], phenazine-1-carboxamide [5], and pyocyanin [6]. In addition to simple phenazines, *Streptomyces* can biosynthesize phenazine derivatives with more complex structures, such as diphenazines, terpenoidal phenazines, carbohydrate-containing phenazines, and saphenic acid derived phenazines [1]. The biological activity of phenazine derivatives varies with the type and number of functional groups attached to the phenazine nucleus [1,7]. For example, PCA showed higher inhibitory activity than 1-hydroxyphenazine against plant disease pathogens such as *Alternaria solani* and *Fusarium oxysporum* [8]. Thus, investigation of side chain modification during the biosynthesis of phenazine derivatives is very important.

In *Streptomyces* and *Pseudomonas*, the biosynthetic process of phenazine products begins with shikimic acid. In following steps, chorismic acid is synthesized through the shikimate pathway then converted into intermediates containing a phenazine nucleus [9]. Two intermediates for all phenazine products, PCA and phenazine-1,6-dicarboxyl acid (PDC), are synthesized via this pathway by the highly conserved *phz* gene cluster [9,10]. The *phzABCDEFG* gene cluster was first sequenced in *Pseudomonas fluorescens* 2–79 [11], and has since been sequenced in many other *Pseudomonas* species, including *Pseudomonas aeruginosa* strains PAO1 [12] and M18 [13], and *Pseudomonas chlororaphis* strains PCL 1391 [14], 30–84 [15], GP72 [16], and 2–79 [11]. The first sequenced phenazine biosynthesis gene cluster in *Streptomyces* was *ephzBCDEGA*, which contributes to the biosynthesis of endophenazines in *Streptomyces cinnamonesis* DSM1042 [17]. Until now, six other *phz* gene clusters have been described in *Streptomyces* [18].

PCA is the core structure for all phenazine biosynthesis products in *Pseudomonas* [19], whereas both PCA [20] and PDC [21] can form the core structure in *Streptomyces*. All phenazine derivatives are further biosynthesized from PCA or PDC by modification of the side chains. The phenazine-modifying genes in *Pseudomonas*, including the *phzM* methyltransferase and *phzS* salicylate hydroxylase genes from *P*. *aeruginosa* [22], and the *phzH* asparagine synthetase gene from *P*. *chlororaphis* PCL1391 [14], have been extensively studied. Because of the complicated structure of phenazine derivatives in *Streptomyces*, only a few genes for phenazine-modifying proteins, such as the prenyltransferase genes, have been examined in *Streptomyces* [9].

Monooxygenases play an important role in oxidation reactions in a number of secondary metabolite biosynthesis pathways in *Streptomyces* [23–25]. A FMN-dependent monooxygenase is involved in dihydrokalafungin oxidation catalysis, the last step in the biosynthesis of the natural antibiotic actinorhodin in *S*. *coelicolor* [26]. A P450 monooxygenase NysL is responsible for C-10 hydroxylation during biosynthesis of the polyene macrolide antibiotic nystatin in *S*. *noursei* [27]. Monooxygenases are also involved in the biosynthesis of phenazine compounds [12,15,16]. A FAD-dependent monooxygenase PhzS from *P*. *aeruginosa* PAO1 catalyzes hydroxylative decarboxylation of PCA to yield 1-OH-Phz [12]. *phzO*, a gene encodes a flavin-dependent aromatic monooxygenase that hydroxylates PCA to produce 2-hydroxyphenazine-1-carboxylic acid (2-OH-PCA), has been found in both *P*. *aureofaciens* 30–84 [15] and *P*. *chlororaphis* GP72 [16]. Until now, no monooxygenase for the hydroxylation of phenazine compounds in *Streptomyces* has been reported.

Lomofungin is an olive-yellow phenazine antibiotic that was first discovered in *Streptomyces lomondensis* sp. n. [28]. This antibiotic has broad-spectrum antibacterial activity against both Gram-positive and Gram-negative bacteria, as well as pathogenic fungi [29–32]. However, despite these advantageous properties, the application of lomofungin has been limited by the low production titer during strain cultivation. *S*. *lomondensis* S015, which can biosynthesize lomofungin, was isolated from rhizosphere soil in Shanghai, China, by our group. We have since worked to improve lomofungin production in this strain, both by optimization of fermentation conditions and by overexpression of regulatory genes [33,34].

In this study, the lomofungin biosynthesis genes were examined after the whole genome sequenc of *S*. *lomodensis* S105 by comparison with available known sequences in *P*. *chlororaphis* GP72 (GenBank: HM594285.1). In addition to the phenazine biosynthesis core gene cluster, a putative flavin-dependent monooxygenase (*lomo10*), responsible for the hydroxylation of lomofungin, was also identified, and was further characterized by in-frame partial deletion.

## Materials and Methods

### 2.1 Bacterial strains, plasmids, and growth conditions

The bacterial strains and plasmids used in this study are described in Table 1 [35,36,37]. The primers used for polymerase chain reaction (PCR) assays are described in Table 2 [36].

**Table 1:** Bacterial strains and plasmids used in this study.

Strains	Characteristics	Reference/source
***Escherichia coli***		
DH5α	Host for general cloning	TranGen Biotech
ET12567 (pUZ8002)	Donor strain for intergeneric conjugation Km^R^, Cm^*R*^	MacNeil et al. [35]
***Streptomyces lomondensis***		
S015	Wild-type, lomofungin-producing strain	Our laboratory
DCC601	Δ*lomo10* mutant	This study
DCC602	*lomo10*-complemented strain DCC601 (containing pIB139-*lomo10*), Apr^r^	This study
**Plasmids**		
pKC1139	*E*. *coli*-*Streptomyces* shuttle vector temperature-sensitive, Apr^r^	Bierman et al. [36]
pIB139	*E*. *coli*-*Streptomyces erythraea* integrative shuttle vector containing a strong constitutive PermE* promoter, Apr^r^	Wilkinson et al. [37]
pMD19-T	General cloning plasmid, Am^r^	Takara Bio
pCC601	Left arm of *lomo10* subcloned into pMD19-T, Am^r^	This study
pCC602	Right arm of *lomo10* subcloned into pMD19-T, Am^r^	This study
pCC603	Left and right arms ligated into pMD19-T, Am^r^	This study
pKC1139-*lomo10*	Plasmid for in-frame partial deletion of *lomo10*, left and right arms ligated into pKC1139 at the *Hin*dIII/*Bgl*II/*Xba*I sites, Apr^r^	This study
pCC604	*lomo10* ligated into pMD19-T, Am^r^	This study
pIB139-*lomo10*	Vector for *lomo10* complementation, Apr^r^	This study

**Table 2:** Primers used in this study.

Primers	Sequence (5′–3′)	Enzyme sites
***lomo10* left arm-For**	AAATTTGAATTCTGGATGATCGCGACGATTTC	*Hin*dIII
***lomo10* left arm-Rev**	AAAAAAAAAAAAATTTAGATCTCATCTCCTGCAGACCCCGAGT	*Bgl*II
***lomo10* right arm-For**	AAATTTAGATCTGAGTTCGTCAAGGTCAGCTCCC	*Bgl*II
***lomo10* right arm-Rev**	AAATTTTCTAGACAGTCGGGGAAGCACTTGAG	*Xba*I
***lomo10*-For**	ACGCATATGGTGGTGCTCGGGGCCAGCATCG	*Nde*I
***lomo10*-Rev**	ACGTCTAGATTCCGATTTCTCAGCGCTGTC	*Xba*I
**pIB-F [36]**	TTGCGCCCGATGCTAGTCG	
**pIB-R [36]**	GCACGACAGGTTTCCCGACTG	

*S*. *lomondensis* S015 (China Center for Type Culture Collection No: M2013140) and its mutants were cultivated at 28°C according to Wang et al. [33]. Seed cultures and fermentations were performed using mannitol soybean (MS) medium (2% mannitol, 2% soybean powder, 2% agar, pH 7.2) and yeast malt (YM) medium (0.4% yeast extract, 1% malt extract, 0.4% glucose, pH 7.2), respectively.

All *Escherichia coli* strains were grown in Luria-Bertani (LB) medium (1% tryptone, 0.5% yeast extract, 1% NaCl, pH 7.2) at 37°C with appropriate antibiotics, as described by Kieser et al. [38].

### 2.2 DNA isolation, manipulation, and sequencing

Genomic DNA was isolated using the method described by Hopwood et al. [38], and DNA was further manipulated according to Maniatis et al. [39,40]. PCR amplicons were isolated from agarose gel using a DNA Gel Extraction Kit (TranGen Biotech, Beijing, China). PCRs were performed in a 25 μl volume using PrimerSTAR HS DNA polymerase (Takara Bio, Dalian, China) with genomic DNA as template. PCR products were purified using an EasyPure PCR Purification Kit (TranGen Biotech). Primers were synthesized by Invitrogen, Shanghai, China. DNA was sequenced by Huada, Shenzhen, China.

### 2.3 Genome and protein sequence analysis

The lomofungin biosynthesis gene cluster was identified from the whole genome sequencing results of *S*. *lomondensis* S015 and analyzed using the antiSmash program (http://antismash.secondarymetabolites.org, accessed on June 25^th^, 2013) [41]. The identified sequence was then aligned with the phenazine biosynthesis gene cluster from *P*. *chlororaphis* GP72 [3,18] for confirmation. The sequences of the surrounding genes were subjected to similarity comparisons and functional predictions using the BLAST program of the NCBI GenBank database (http://blast.ncbi.nlm.nih.gov/Blast.cgi). The website was accessed on April 10^th^, 2013.

### 2.4 Construction of the *lomo10* deletion mutant strain DCC601

The *lomo10* gene was disrupted using the pKC1139-*lomo10* inactivation plasmid. Two flanking regions or “arms” (1,996 and 1,784 bp), containing the upstream and downstream regions of *lomo10*, were amplified by PCR (30 cycles, 98°C for 10 s, 55°C for 15 s, and 68°C for 2 min) from *S*. *lomondensis* S015 genomic DNA using the primers *lomo10* left arm For/Rev and *lomo10* right arm For/Rev, respectively. The resulting products were individually ligated into the TA cloning vector pMD19-T to yield pCC601 and pCC602, respectively. Both insertions were verified by DNA sequencing. The downstream fragment from pCC602 was then excised using *Hin*dIII and *Bgl*II and ligated into the corresponding restriction sites in pCC601 to generate pCC603. The complete 3780 bp fragment from pCC603 was then excised and ligated to the corresponding *Hin*dIII and *Xba*I sites of pKC1139, generating inactivation plasmid pKC1139-*lomo10*.

pKC1139-*lomo10* was first introduced into *E*. *coli* ET12567(pUZ8002) via heat shock transformation to generate the donor strain, then introduced into *S*. *lomondensis* S015 by conjugation [42]. Following incubation of the transconjugants at 28°C for 18 h, 1 ml of sterile water containing nalidixic acid and apramycin, both at a final concentration of 50 μg/ml, was spread onto the surface of the MS plate. Transconjugants were incubated for a further 2–4 days at 28°C, and resulting colonies were streaked onto solid MS medium containing 50 μg/ml apramycin at 37°C to yield single-crossover homologous recombination mutants. To inactivate *lomo10*, single crossover mutants were cultured at 37°C with shaking at 220 rpm for 3 days in a 250 ml flask containing 50 ml YM liquid medium. These mutants were cultured for five successive generations without apramycin to generate a double cross-over mutant that was sensitive to apramycin. In-frame deletion of *lomo10* in the resulting positive mutant was confirmed by PCR (30 cycles, 98°C for 10 s, 55°C for 15 s, and 68°C for 2 min) with primers *lomo10* left arm-For and *lomo10* right arm-Rev.

### 2.5 Construction of the *lomo10* self-complementation strain DCC602

The 1,326 bp *lomo10* region was amplified (30 cycles, 98°C for 10 s, 55°C for 15 s, and 68°C for 1 min) from *S*. *lomondensis* S015 genomic DNA using the primers *lomo10*-For and *lomo10*-Rev. The PCR product was gel-purified and ligated into pMD19-T to form plasmid pCC604, which was confirmed by DNA sequencing. The *lomo10* fragment was then excised from pCC604 using restriction enzymes *Nde*I/*Xba*I, and ligated into the corresponding sites of the integrative vector pIB139 [37] to yield the self-complementary plasmid pIB139-*lomo10*. pIB139-*lomo10* was then introduced into the *S*. *lomondensis* S015 *lomo10* deletion mutant strain, DCC601, by conjugation from *E*. *coli* ET12567(pUZ8002). A positive exconjugant was obtained by apramycin resistance screening and confirmed by PCR amplification and DNA sequencing using the primer pair pIB-F/pIB-R [43] and the thermal cycler parameters mentioned above.

### 2.6 High performance liquid chromatography (HPLC) analysis

Phenazine products in wild-type S015, *lomo10*-inactivated mutant DCC601, and *lomo10*-complemented strain DC602 were analyzed by HPLC as described by Wang et al. [33]. For sampling, up to 5 ml of culture broth were centrifuged at 10,800 × *g* for 8 min. The supernatant was adjusted to pH 2.0 using an aqueous HCl solution (6 M), and mixed with 5 ml of pure butanone. The resulting mixture was centrifuged at 10,800 × *g* for 5 min and the upper layer was collected. The water layer (lower layer) was extracted a second time using 5 ml of pure butanone, and the combined extracts were dried using a rotary vacuum dryer (Christ RVC 2–18, Osterode, Germany) at 33°C. The resulting residue was dissolved in 5 ml of HPLC grade solvent, a 1:1 (v/v) mixture of 0.1% formic acid and acetonitrile, and filtered through a 0.22 μm polyvinylidene difluoride syringe filter (Millipore, Shanghai, China). A 20:l sample of the resulting filtrate was analyzed by HPLC using an Agilent 1260 HPLC system (Agilent, Beijing, China) equipped with a DAD detector and an Agilent Eclipse Plus C18 column (250 × 4.6 mm; 5 μm), which was used at 30°C. The mobile phase consisted of solvents A (0.1% formic acid) and B (acetonitrile), which were used with the following gradient profile: 0–4 min, 80–60% A; 4–20 min, 60% A; and 20–30 min, 60–80% A. The HPLC system was operated at a constant flow rate of 1 ml/min.

### 2.7 Purification and structural analysis of the new phenazine product

Mutant DCC601 was used for large-scale fermentation (10 l) in yeast extract-malt extract broth at 28°C and 180 rpm for 96 h [33]. The liquid culture was centrifuged at 7,104 × *g* for 30 min, and then adjusted to pH 2.0 using an aqueous HCl solution (6 M). The supernatant was extracted four times with 2.5 l butanone. All four supernatants were combined and then concentrated using a vacuum evaporator at 33°C to remove the organic phase. For further purification, the raw extract was dissolved in 0.1% formic acid/acetonitrile (1:1, v/v) and purified by preparative HPLC using an Agilent 1200 series apparatus with a C18 column (ZOBRAX-C18 column, 5 μm, 10.0 × 250 mm, Elite, Dalian, China). The mobile phase consisted of 0.1% formic acid/acetonitrile (60:40, v/v) with a flow rate of 1 ml/min (detection by absorbance at 270 nm). The peak containing the pure compound was collected and dried under vacuum and freezing.

The dried intermediate metabolite was then used for further liquid chromatography-high resolution mass spectrometery (LC-HRMS) and nuclear magnetic resonance (NMR) analyses. The LC-HRMS was performed on a Waters ACQUITY UPLC system (Waters Corporation, Milford, MA) with an ACQUITY BEH C18 column (100 mm × 2.1 mm, 1.7 μm; Waters). The column was eluted with gradient solvent of 20:80 solutions B:A (0–15 min), 40:60 solutions B:A (15.01–25 min), and 20:80 solutions B:A (25.01–35 min) at a flow rate of 0.40 ml/min, where solution A is 0.1% (v/v) formic acid and solution B is acetonitrile. The system was monitored by measuring UV absorbance at 270 nm. The mass spectrometer was run in positive ion detection mode set to scan between 50 and 1,000 m/z. The NMR assay was performed with a Bruker NMR spectrometer (Avance III 600 MHz; Bruker, Karlsruhe, Germany).

## Results

### Analysis of lomofungin biosynthesis genes based on whole genome sequence of *S*. *lomondensis* S015

**Fig 1:**
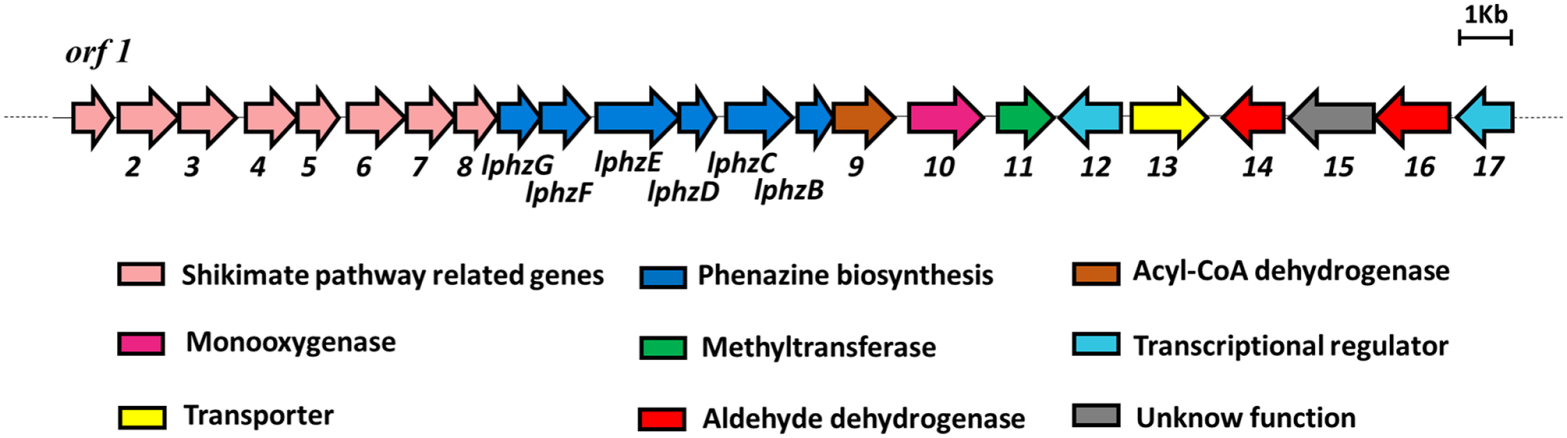
Putative lomofungin biosynthesis gene cluster in *Streptomyces lomondensis* S015.

Sequencing and assembly of the *S*. *lomondensis* S015 genome resulted in a draft genome size of 9,448,526 bp and a GC content of 71.7%. The antiSmash program and alignment of the whole genome sequence with the phenazine biosynthesis gene cluster of *P*. *chlororaphis* GP72 [3,18] identified a putative gene cluster for the biosynthesis of lomofungin. This contiguous DNA region contains 23 open reading frames (Fig 1, Table 3). Six of the genes, designated *lphzGFEDCB*, showed high similarity (53.4–72.2%) at the amino acid level to the phenazine biosynthesis genes *phzBCDEFG* of *P*. *chlororaphis* GP72 [3,18]. Except for the absence of a *phzA* ortholog, and the order of the genes, the high similarity of these six genes to the corresponding genes in *P*. *chlororaphis* GP72 suggested a close evolutionary relationship with this species.

**Table 3:** Genes in the putative lomofungin biosynthesis cluster.

ORFs	aa	Proposed function	Identity (%)	Ortholog identified by BLAST search
*lomo1*	201	Shikimate kinase	88%	*Streptomyces fulvoviolaceus*
*lomo2*	378	Chorismate synthase	97%	*Streptomyces fulvoviolaceus*
*lomo3*	272	3-phosphoshikimate- 1-carboxyvinyltransferase	78%	*Streptomyces* sp. NRRL B-5680
*lomo4*	306	5,10-methylenetetrahydrofolate reductase	90%	*Streptomyces* sp. NRRL S-646
*lomo5*	203	Hexokinase	71%	*Streptomyces albaduncus*
*lomo6*	398	S-adenosylmethionine synthetase	90%	*Streptomyces* sp. NRRL S-646
*lomo7*	201	Quinone oxidoreductase	78%	*Streptomyces anulatus*
*lomo8*	162	Flavin reductase	85%	*Streptomyces* sp. NRRL S-646
*lphzG*	213	*phzG*	53%	*Pseudomonas chlororaphis* GP72
*lphzF*	278	*phzF*	66%	*Pseudomonas chlororaphis* GP72
*lphzE*	598	*phzE*	63%	*Pseudomonas chlororaphis* GP72
*lphzD*	111	*phzD*	65%	*Pseudomonas chlororaphis* GP72
*lphzC*	368	*phzC*	60%	*Pseudomonas chlororaphis* GP72
*lphzB*	160	*phzB*	72%	*Pseudomonas chlororaphis* GP72
*lomo9*	354	Acyl-CoA dehydrogenase	95%	*Streptomyces* sp. NRRL S-646
*lomo10*	442	Flavin-dependent monooxygenase	48%	*Streptomyces viridosporus*
*lomo11*	273	Methyltransferase	39%	*Streptomyces auratus*
*lomo12*	342	Transcriptional regulator	94%	*Streptomyces* sp. NRRL S-646
*lomo13*	484	Transporter	91%	*Streptomyces* sp. NRRL S-646
*lomo14*	314	Alcohol dehydrogenase	89%	*Streptomyces* sp. NRRL S-646
*lomo15*	340	EsmB1, phenazine antibiotic biosynthesis protein	72%	*Streptomyces antibioticus*
*lomo16*	435	Aldehyde dehydrogenase	86%	*Streptomyces* sp. NRRL S-646
*lomo17*	337	Transcriptional regulator	86%	*Streptomyces* sp. NRRL S-646

Sequences from *Streptomyces lomondensis* S015 have been deposited in the NCBI GenBank database under accession number KP721214.

A set of eight genes, designated *lomo1*–*8*, were most likely to code for enzymes relating to the shikimate pathway [2]. *lomo9* showed 95% amino acid similarity to the acyl-CoA dehydrogenase gene from *Streptomyces sp*. NRRL S-646, which oxidizes branched-chain acyl-CoA fatty acid derivatives and macrolide antibiotics in *Streptomyces coelicolor* and *Streptomyces avermitilis* [44]. *lomo10* showed 95% amino acid similarity to monooxygenases genes from *Streptomyces acidiscabies* and *Streptomyces noursei*, which are responsible for hydroxylation during the biosynthesis of thaxtomin A [9] and the polyene macrolide antibiotic nystatin, respectively [27,45]. *lomo11* codes for a protein with moderate similarity (39%) to a putative methyltransferase that has been reported to catalyze the conversion of macrocin to tylosin [46]. Both *lomo12* and *lomo17* showed high similarity to the transcriptional regulatory genes in *Streptomyces* sp. NRRL S-646 and they both belong to the AsnC family.

### Inactivation and self-complementation of *lomo10*

**Fig 2:**
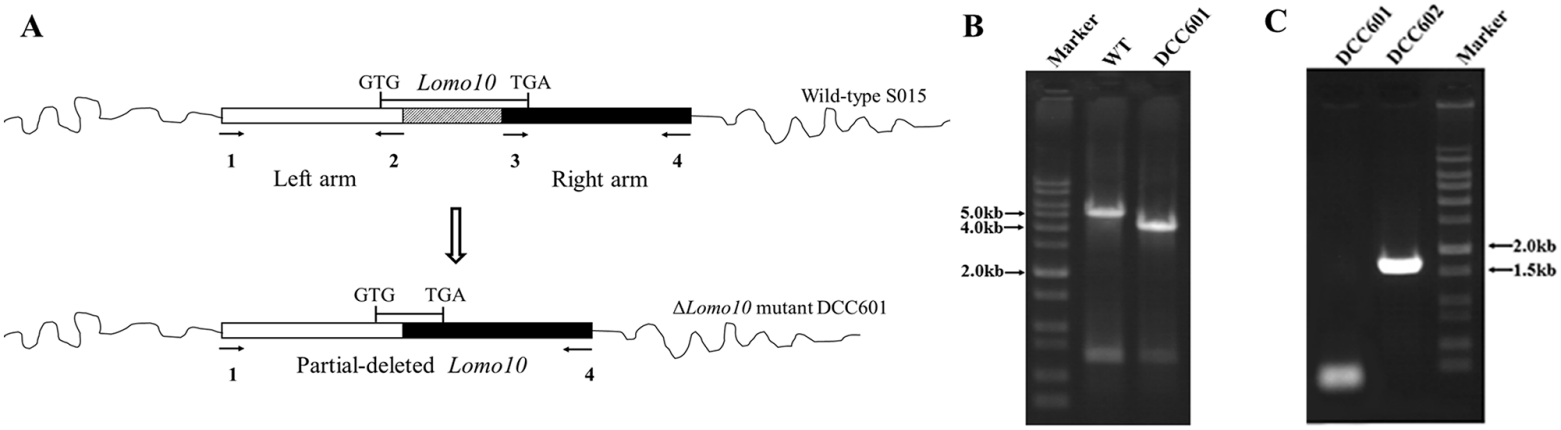
Inactivation and self-complementation of *lomo10* in *Streptomyces lomondensis* S015. (A) Schematic of the in-frame partial deletion of 1032 bp in *lomo10* to generate the Δ*lomo10* mutant DCC601. Primers 1, 2, 3, and 4 were used to amplify the left and right homology arms. GTG and TGA were the start and termination codons for *lomo10*, respectively. The expected PCR product from the wild-type (WT) strain was 5,106 bp, and that from DCC601 was 4,074 bp using the primers *lomo10* left arm-For and *lomo10* right arm-Rev. (B) PCR analysis of WT strain and DCC601. (C) PCR analysis of DCC601 and *lomo10* complementation strain DCC602. The amplicon generated from the DCC602 genomic DNA gave the expected 1,726 bp fragment, but no band was amplified from the DCC601 strain.

**Fig 3:**
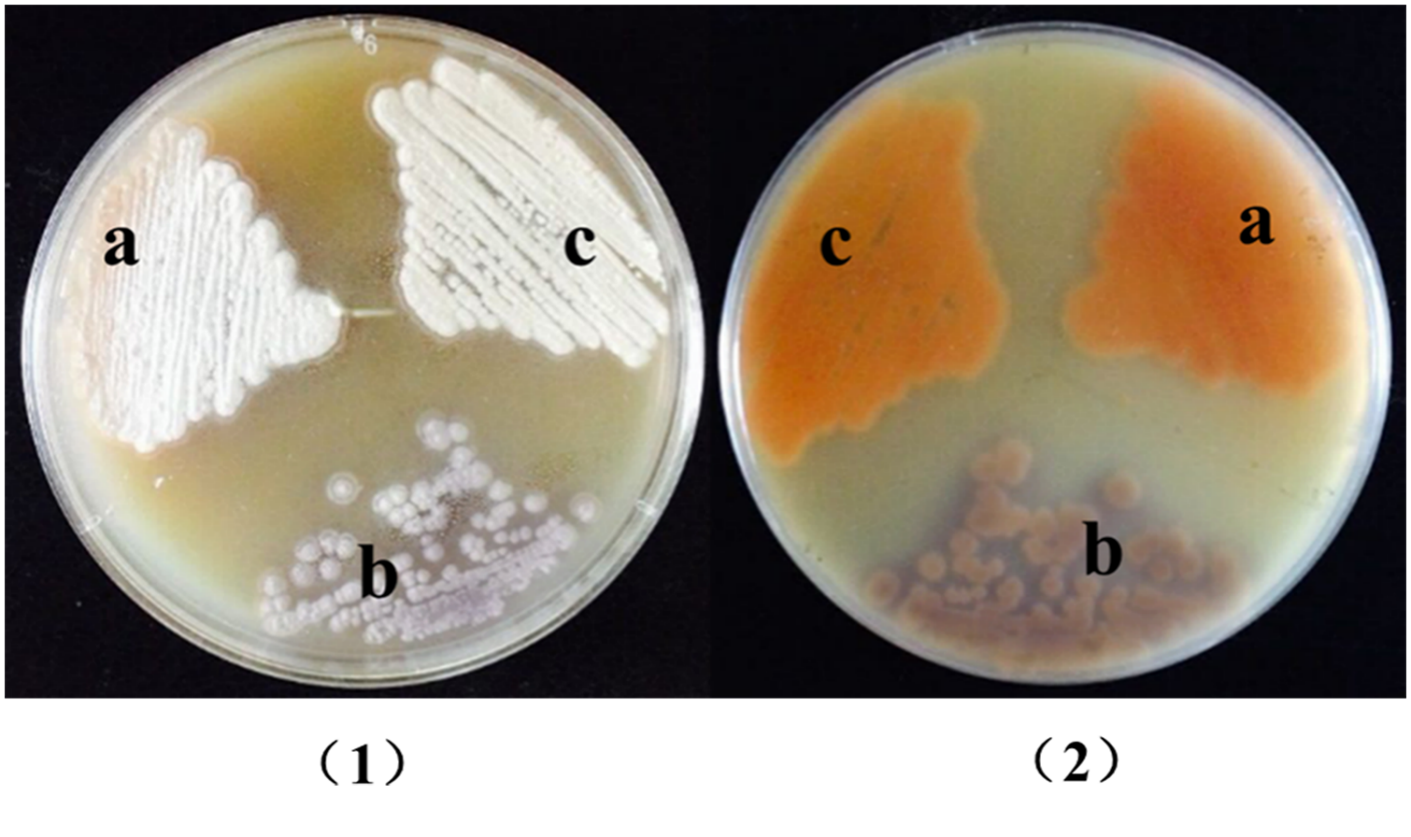
Photographs of the different strains grown on MS medium. (A) Wild-type strain. (B) *lomo10* inactivated mutant DCC601. (C) *lomo10* complementation strain DCC602.

As there are three hydroxyl groups included in the structure of lomofungin, the function of the putative hydroxylation gene *lomo10* was investigated by generating an in-frame partial deletion mutant, DCC601, via double-crossover homologous recombination (Fig 2A). Strain DCC601 was obtained by deletion of the 1032 bp in the coding region of *lomo10*. No promoter was found in this region by using online software (http://www.fruitfly.org/seq_tools/promoter.html) analysis. Thus, the partial deletion of *lomo10* might not affect the expression of other genes, such as *lomo11*. The genotype of the DCC601 *lomo10* deletion mutant, as well as that of the *lomo10* self-complementation strain DCC602, was confirmed by PCR analysis as shown in Fig 2B and 2C, respectively and DNA sequencing. The phenotypes of wild-type strain S015, DCC601, and DCC602 were examined following culture on solid MS medium (Fig 3). Wild-type strain S015 produced the olive-yellow lomofungin (Fig 3A), whereas the *lomo10* deletion mutant (DCC601) produced light purple colored colonies (Fig 3B). Complementation with *lomo10* restored lomofungin production (Fig 3C). There was no obvious difference in the phenotype of mycelium between WT, mutant strain DCC601 and complementary strain DCC602.

**Fig 4:**
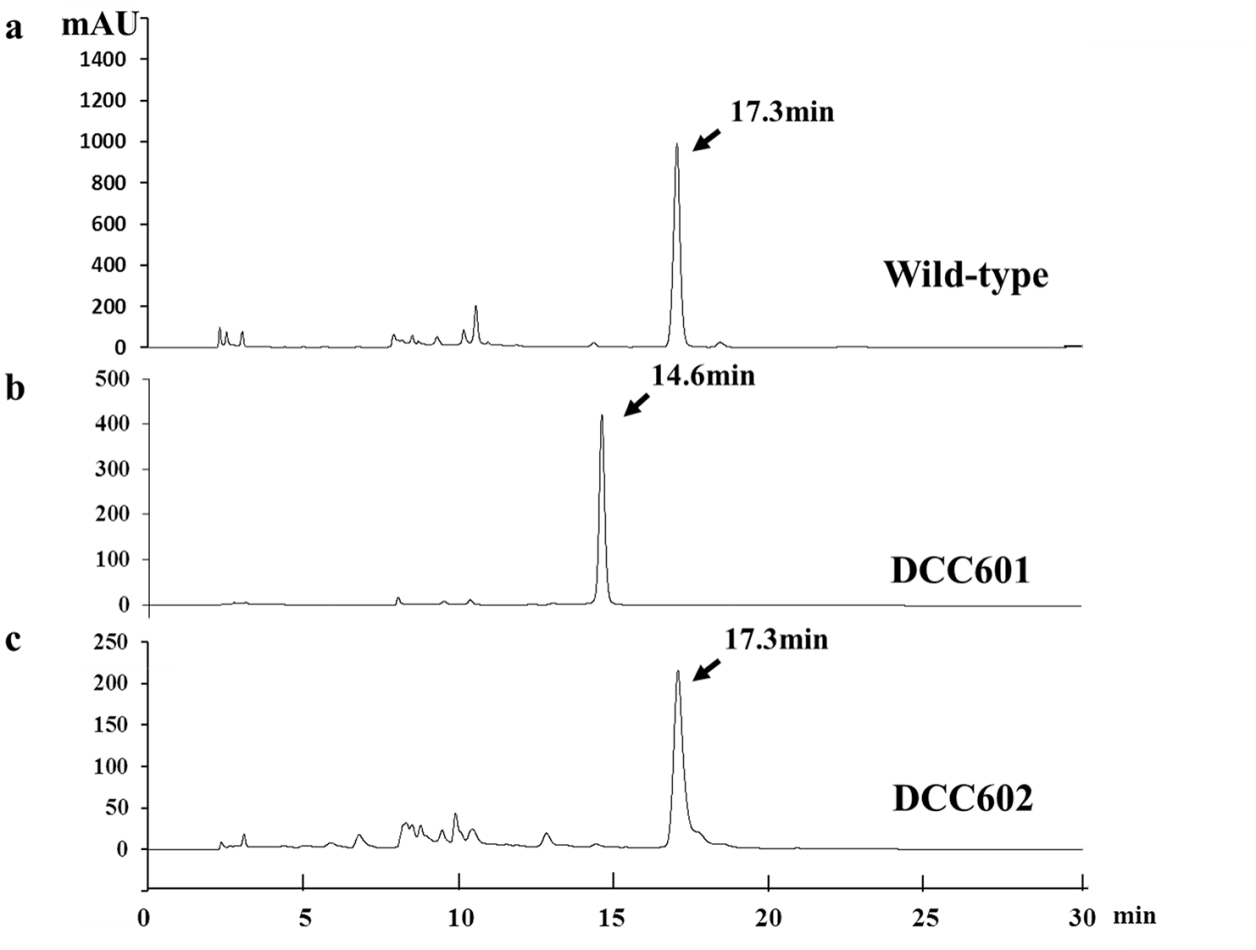
HPLC profiles of fermentation products from different strains. (A) Wild-type strain. (B) *lomo10* inactivated mutant DCC601. (C) *lomo10* complementation strain DCC602. lomofungin (Rt = 17.3 min), compound A (Rt = 14.6 min).

The HPLC profiles of the fermentation products of wild-type S015, DCC601, and DCC602 are illustrated in Fig 4. The peak corresponding to lomofungin appeared at a retention time of 17.3 min in the wild-type strain, but was eliminated in the knockout mutant, and a new peak with a retention time of 14.6 min appeared. Comparison of the HPLC profiles of the fermentation products of extracts from complementation strain DCC602 showed that lomofungin production had been restored in this strain. The new compound produced by the mutant strain, compound A, was purified, and its structure was characterized by LC-HRMS and NMR analyses.

The methods and results of whole-cell biotransformation of compound A by Lomo10 were shown in S1 File and S1 Fig, respectively. After 2 h reaction, lomofungin was synthesized in the reaction system of *E*.*coli* DH5μ/pMD-18T-*lomo10* mixed with compound A. The results further verified that Lomo10 could hydroxylates compound A to produce lomofungin.

### Purification and structural analysis of compound A

**Fig 5:**
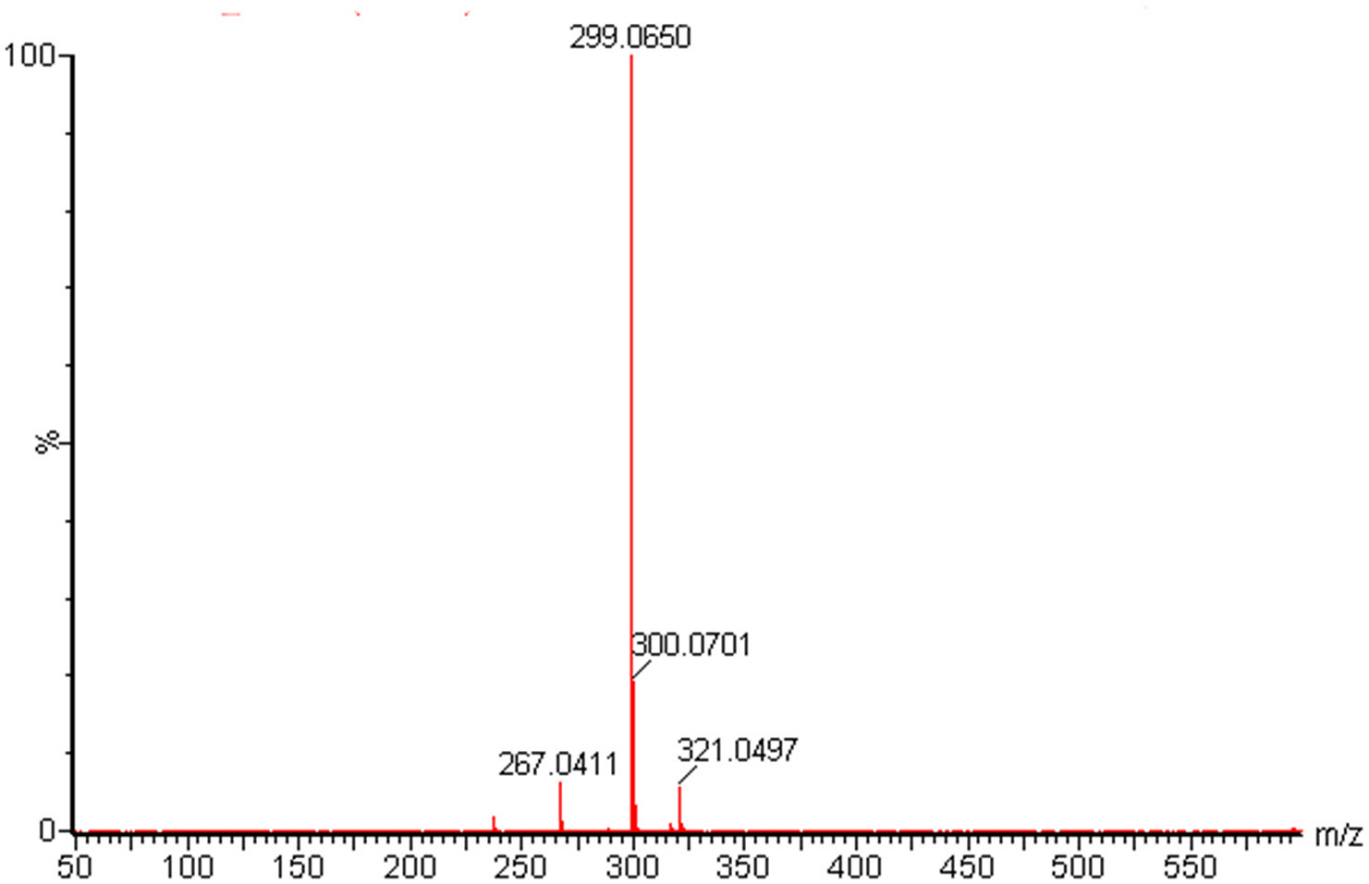
Spectrum of compound A following LC-HRMS analysis. The caculated mass of compound 1 was determined to be 298.25 (m/z 299.07).

**Fig 6:**
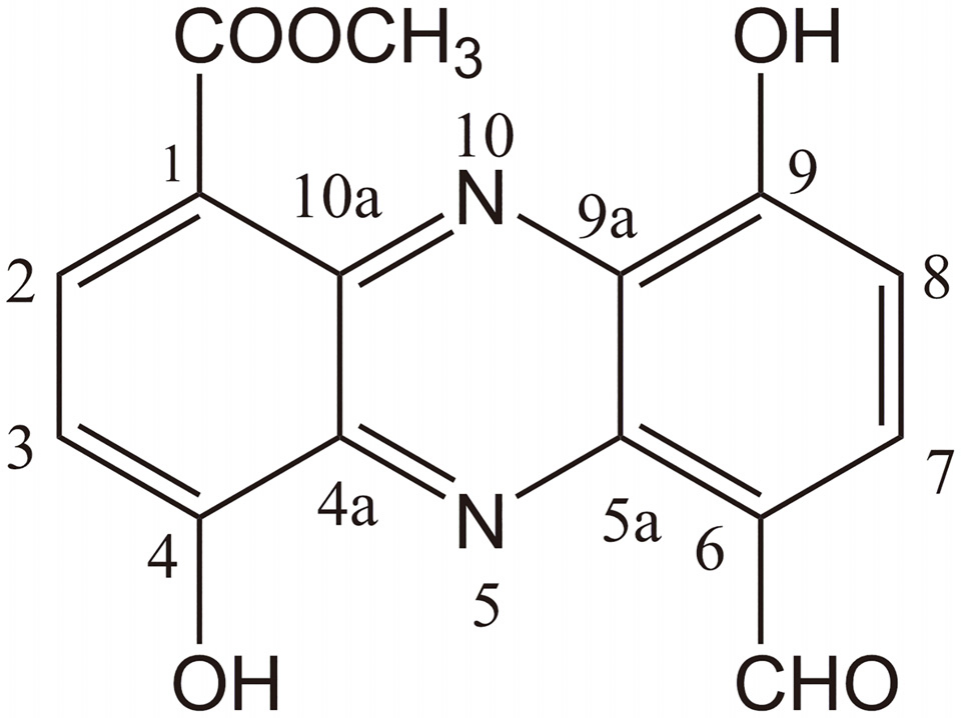
Chemical structure of compound A.

To confirm the structure of the new compound, compound A was purified from 10 l of fermentation broth. In total, 80 mg of compound A was obtained following purification. The exact mass of compound A obtained from LC-HRMS was 299.07 for [C_15_H_10_N_2_O_5_]^+^ (calculated mass, 298.25; Fig 5). This calculated mass was 16 Da smaller than the known mass of lomofungin, suggesting that compound A contained one less hydroxyl than lomofungin (C_15_H_10_N_2_O_6_, MW 314 Da) [47]. Structural analysis was performed by ^1^H NMR, ^13^C NMR, distortionless enhancement by polarization transfer-90, ^1^H-^1^H correlation spectroscopy, heteronuclear multiple bond correlation, and heteronuclear multiple quantum coherence analyses, and the proton and carbon chemical shifts of compound A are shown in Table 4 and in S2 Fig, respectively. The ^1^H-NMR spectra contained four proton signals (δ_H_, 7.39–8.41 ppm) that are typical of double bonds (ring hydrogen), along with corresponding ^13^C-NMR spectra of carbon atoms (δ_C_, 110.1–134.5 ppm), suggesting that compound A has a phenazine ring in the core of its structure. Compared with the reported NMR data for lomofungin [47], the main differences were the absence of a hydroxyl hydrogen at δ_H_ 11.22, and the presence of hydrogen ring at δ_H_ 8.27. Therefore, compound A was predicted to be 1-carbomethoxy-6-formy-4,9-dihydroxy phenazine, a new chemical compound missing the C-7 hydroxyl of lomofungin (Fig 6).

**Table 4:** Chemical shift summarized from ^1^H (DMSO-d_6_) and ^13^C (MeOD-d_6_) analyses recorded by 600 MHz NMR spectrometry.

^1^H NMR	Chemical shift δ (ppm)	^13^C NMR	Chemical shift δ (ppm)
-COOCH_3_	3.95, s	C-1	120.9
C-2	8.41, d	C-2	134.4
C-3	7.30, d	C-3	111.2
C-7	8.28, d	C-4	156.5
C-8	7.39, d	C-4a	141.3
-CHO	11.40,s	C-5a	135.2
		C-6	123.0
		C-7	134.5
		C-8	110.1
		C-9	160.2
		C-9a	133.4
		C-10a	139.8
		-CHO	190.7
		-COOCH_3_	52.1
		-COOCH_3_	166.2

## Discussion

Phenazine biosynthesis gene clusters are normally composed of seven genes arranged in order (e.g. *phzABCDEFG*), and have been located downstream of shikimate pathway-related genes in *Pseudomonas* species including *P*. *fluorescens* 2–79, *P*. *aureofaciens* 30–84, and *P*. *chlororaphis* PCL1391 [11,14,15]. Two nearly identical core *phz* gene clusters, called *phzA1-G1* and *phzA2-G2*, with different promoters and flanking regions, have also been found in *Pseudomonas* sp. M18 [48] and *P*. *aeruginosa* PAO1 [12].

Compared with *Pseudomonas*, the number and order of genes within the phenazine biosynthetic gene cluster in *Streptomyces* are more varied. *Streptomyces anulatus* 9663 also has a seven-gene cluster for the biosynthesis of endophenazine A and endophenazine B, but unlike the corresponding region in *Pseudomonas* species, *phzA* is located at the end of the cluster: *ppzBCDEFGA* [9,49]. A six-gene cluster for prenylated phenazine biosynthesis in *Streptomyces cinnamonensis* DSM 1042 is ordered *ephzBCDEGA*, and completely lacks the gene encoding the PhzF protein [17]. Another gene cluster consisting of just *epzAGFC* was identified in *S*. *cinnamonensis* DSM 1042, and is also involved in prenylated phenazine biosynthesis [50]. The whole genome sequencing performed in the current study showed that the phenazine biosynthetic gene cluster in *S*. *lomondensis* S015 is structured *lphzGFEDCB*, and while still located downstream of the shikimate pathway-related genes, the gene order is completely reversed compared with the cluster in *Pseudomonas*, and lacks *phzA*. In addition, we found another two phenazine biosynthesis-related genes, *phzC2* and *phzE2*, in another scaffold of the whole genome of *S*. *lomondensis* S015. Further study is needed to confirm whether they are involved in the biosynthesis of lomofungin.

Until now, monooxygenases for the hydroxylation of phenazine compounds have been identified only in *Pseudomonas*. Most of these monooxygenases use PCA as their substrate [12,15,16]. In the current study, alignment of the *lomo10* amino acid sequence showed that *lomo10* encodes a putative flavin-dependent monooxygenase. Inactivation of *lomo10* in *S*. *lomondensis* S015 resulted in the production of a novel phenazine product containing a deletion of the hydroxyl at the C-7 position of lomofungin. The four monooxygenases that catalyzed the hydroxylation of phenazines were compared and analyzed using DNASTAR Lasergene.v7.1 software and the results were shown in S3 Fig The monooxygenases in *P*. *aureofaciens* 30–84 and *P*. *chlororaphis* GP72 showed very high similarity and they both catalyze the hydroxylation of PCA at its C-2 position. The monooxygenase in *S*. *lomondensis* S015 showed low similarity to others might due to the differences in substrate structure and hydroxylation position because it hydroxylates compound A at its C-7 position.

There are three hydroxyl groups in lomofungin, located at the C-4, C-7, and C-9 positions. Only the C-7 hydroxyl was deleted in the *lomo10* mutant strain DCC601, suggesting that there might be other hydroxylation genes in *S*. *lomondensis* S015. Two P450 monooxygenases, named *lomo56* and *lomo57*, were located downstream of the lomofungin biosynthesis gene cluster in *S*. *lomondensis* S015. These two genes may be involved in the transfer of the C-4 and C-7 hydroxyl groups of lomofungin.

**Fig 7:**
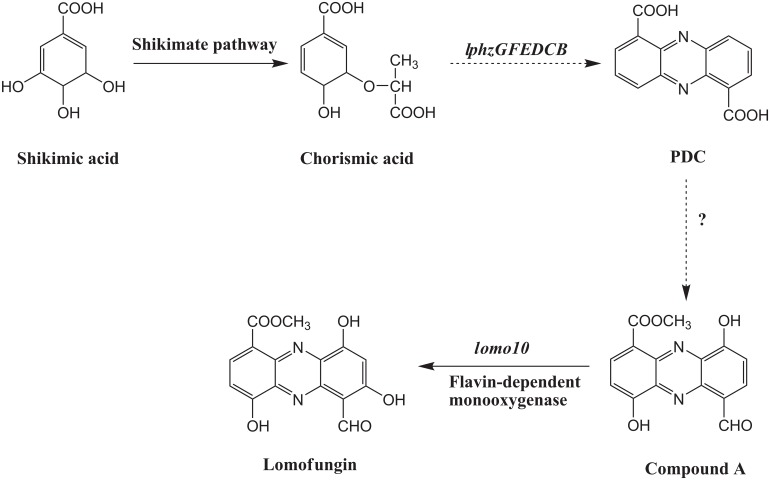
Proposed lomofungin biosynthetic pathway in *Streptomyces lomondensis* S015.

Based on the alignment of the *S*. *lomondensis* S015 genes and the other results obtained in this study, a putative lomofungin biosynthesis pathway is shown in Fig 7. Buckland et al. [51] confirmed that lomofungin is biosynthesized from PDC. Thus, here we propose that the *lphzGFDECB* gene cluster can biosynthesize PDC. The new compound obtained in this study, 1-carbomethoxy-6-formyl-4,9-dihydroxy-phenazine (compound A), might be synthesized from PDC in several steps catalyzed by Lomo9 and Lomo11 et al., and then converted into lomofungin by *lomo10*. Further validation of this pathway is required.

## Supporting Information

S1 Fig HPLC profiles of whole-cell biotransformation of compound A by *E*.*coli* DH5μ/pMD-18T-*lomo10*. (A) Lomofungin standard. (B) Biotransformation system with Lomo10. (C) Control without Lomo10.
(TIF)

S2 Fig NMR spectra of Compound A. (A) ^1^H NMR spectrum in DMSO-d6. (B) ^13^C NMR spectrum in MeOD. (C) COSY spectrum in DMSO-d6. (D) HMBC spectrum in DMSO-d6.
(TIF)

S3 Fig Amino acid blast of monooxygenases among different stains. (TIF)

S1 File Method of biotransformation of compound A. (PDF)
