# Supplementary figures and images for: Identification of the Lomofungin Biosynthesis Gene Cluster and Associated Flavin-Dependent Monooxygenase Gene in Streptomyces lomondensis S015
Source: PLoS One. 2015 Aug 25;10(8):e0136228. doi: 10.1371/journal.pone.0136228 (PMC4549113; doi:10.1371/journal.pone.0136228)

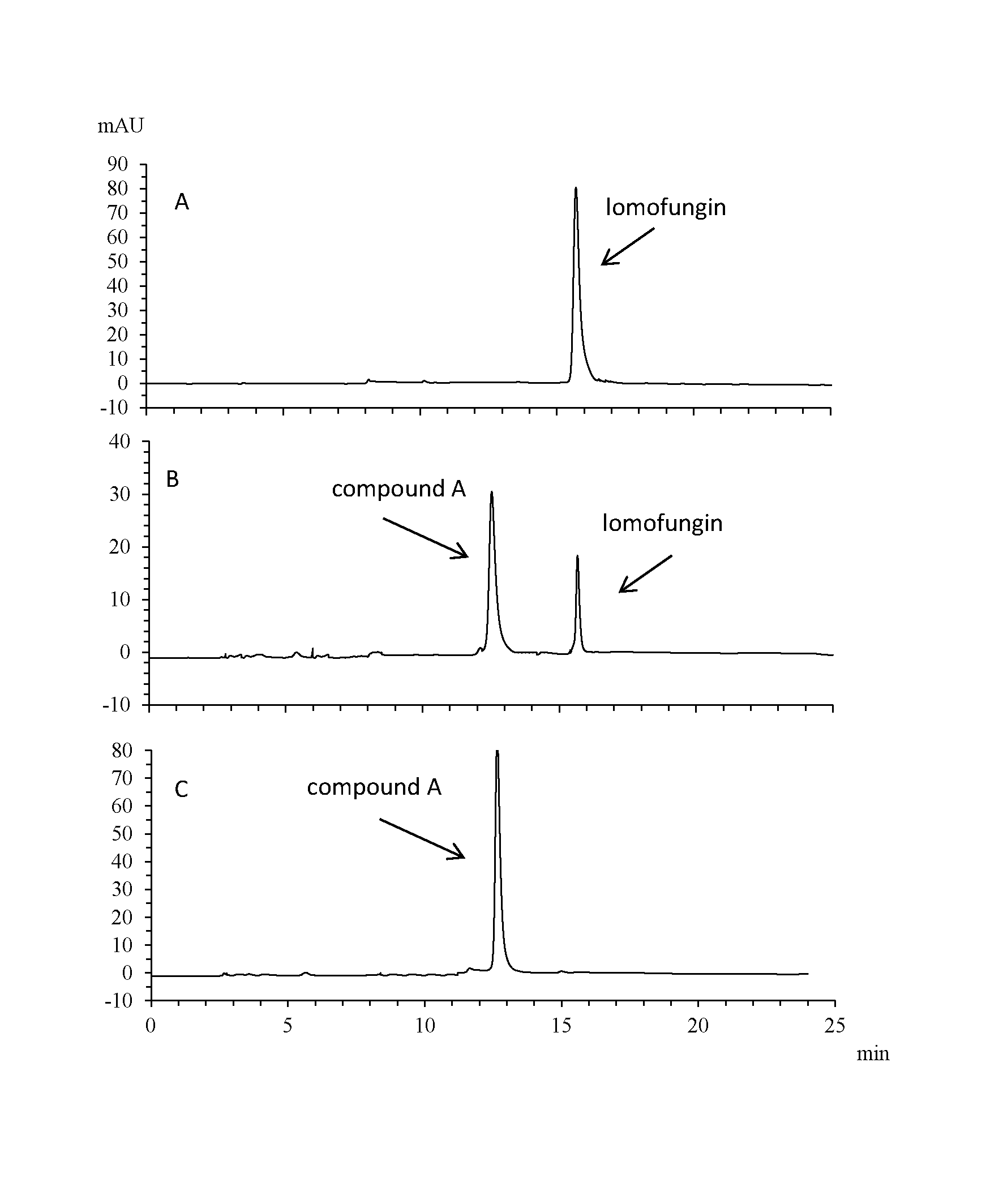

Supplement: S1 Fig — (A) Lomofungin standard. (B) Biotransformation system with Lomo10. (C) Control without Lomo10. (TIF) [file pone.0136228.s001.tif]

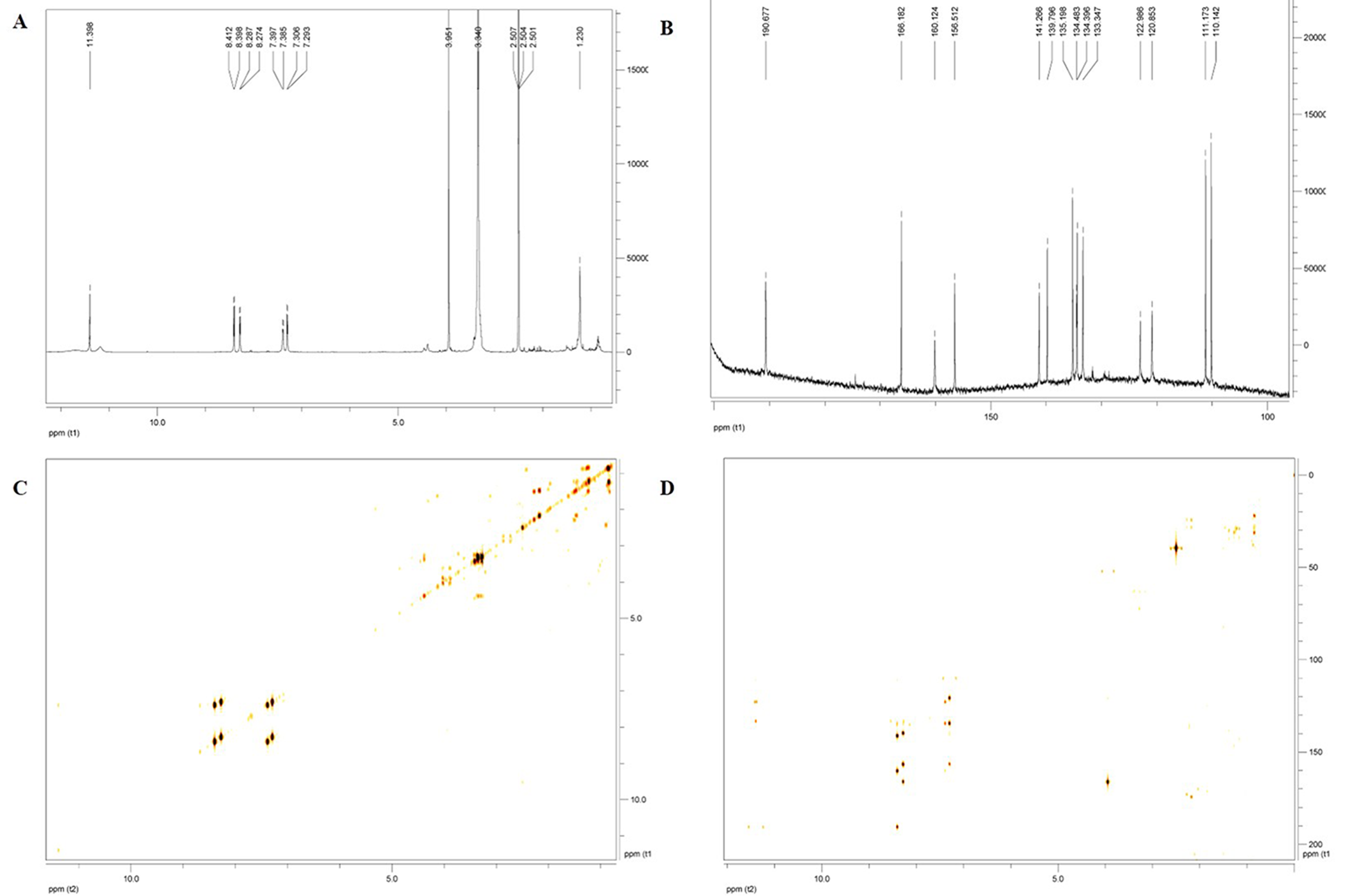

Supplement: S2 Fig — (A) 1H NMR spectrum in DMSO-d6. (B) 13C NMR spectrum in MeOD. (C) COSY spectrum in DMSO-d6. (D) HMBC spectrum in DMSO-d6. (TIF) [file pone.0136228.s002.tif]

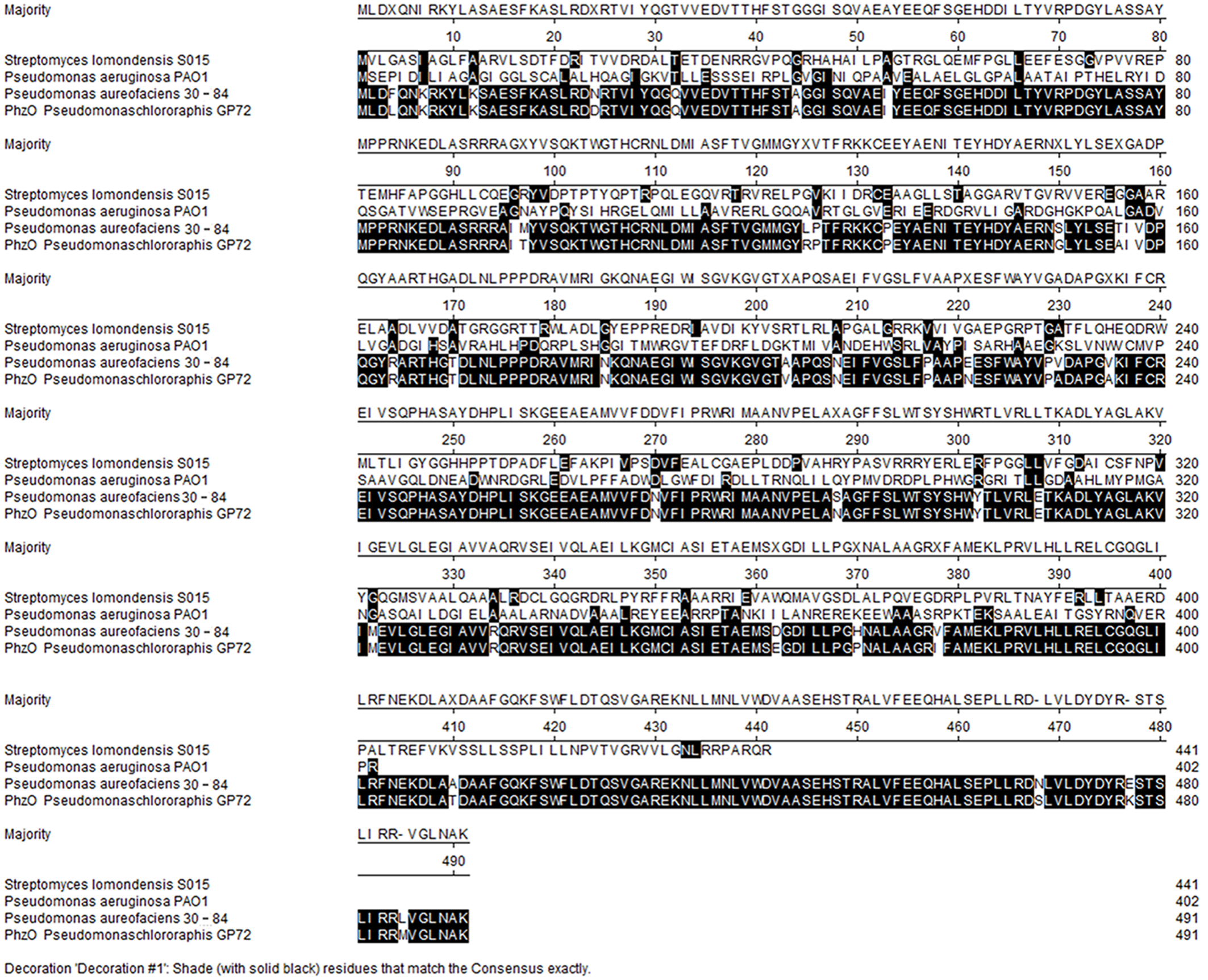

Supplement: S3 Fig — (TIF) [file pone.0136228.s003.tif]
