# Supplementary material for: Identification of the Lomofungin Biosynthesis Gene Cluster and Associated Flavin-Dependent Monooxygenase Gene in Streptomyces lomondensis S015
Source: PLoS One. 2015 Aug 25;10(8):e0136228. doi: 10.1371/journal.pone.0136228 (PMC4549113; doi:10.1371/journal.pone.0136228)
Supplement: S1 File — (PDF) [file pone.0136228.s004.pdf]

Method of biotransformation of compound A

The whole-cell biotransformation of compound A by Lomo10 was done using the method as described by Liu et al [1].

A 1,380 bp *lomo10* gene was amplified from *S. lomondensis* S015 genomic DNA using the primers (F: ATGGTGCTCGGGGCCAGCAT and R: CAGCTTTCAGCCACGCGGAGC). The *lomo10* fragment was ligated into pMD18-T vector (Takara Bio, Dalian, China) to produce pMD18-T-*lomo10*, and then introduced into *E.coli* DH5α for *lomo10* expression. Recombinant *E. coli* cells were grown in LB medium at 37 ℃ to an optical density at 600 nm (OD_{600}) of 0.6 and then induced with 0.8 mM isopropyl-β-D-thiogalactopyranoside (IPTG) for 12 h at 16 ℃.

E. coli DH5α/pMD18-T-*lomo10* cells were harvested by centrifugation, washed, and resuspended in 10 ml phosphate buffer (50 mM, pH 7.4) to a final OD_{600} of 2.0. For qualitative analysis, compound A was added to recombinant *E. coli* cell suspensions at a final concentration of 50 mg/ml. Cell suspensions were incubated with shaking (180 rpm, 28 ℃). After 2 h, the supernatant of the reaction mixture was adjusted to pH 2.0 using an aqueous HCl solution (6 M) and extracted with butanone for HPLC analysis. The *E. coli* DH5α/pMD18-T without *lomo10* gene was constructed, cultivated, induced, mixed with compound A as the control.

An Agilent 1260 HPLC system (Agilent, Beijing, China) equipped with a DAD detector and an Agilent Eclipse Plus C18 column (250 × 4.6 mm; 5 μm) were used at 30 ℃ for HPLC analysis. The mobile phase consisted of 60% solvents A (0.1% formic acid) and 40% acetonitrile.

References

1. Liu H, Wang SJ, Zhang JJ, Dai H, Tang H, Zhou NY. Patchwork assembly of *nag*-like nitroarene dioxygenase genes and the 3-chlorocatechol degradation cluster for evolution of the 2-chloronitrobenzene catabolism pathway in *Pseudomonas stutzeri* ZWLR2-1. Appl Environmental Microbiol 2011; 77: 4547–4552.
